# Protective Effect of Curcumin in Oxidative Stress-Induced Injury on Retinal Pigment Epithelial Cells

**DOI:** 10.3390/jcm14207153

**Published:** 2025-10-10

**Authors:** Hyo Seon Yu, Heeyoon Cho, Yong Un Shin, Eun Hee Hong, Seong-Ho Koh

**Affiliations:** 1 Department of Ophthalmology, Hanyang University College of Medicine, Seoul 04763, Republic of Korea; hello4144@hanyang.ac.kr (H.S.Y.); yushin@hanyang.ac.kr (Y.U.S.); 2 NOON Eye Clinic, Guri 11926, Gyeonggi-do, Republic of Korea; drhycho@gmail.com; 3 Research Institute of Regenerative Medicine and Stem Cells, Hanyang University, Seoul 04763, Republic of Korea; 4 Department of Ophthalmology, Hanyang University Guri Hospital, Guri 11923, Gyeonggi-do, Republic of Korea; 5 Hanyang Institute of Bioscience and Biotechnology, Hanyang University, Seoul 04763, Republic of Korea; 6 Department of Neurology, Hanyang University College of Medicine, Seoul 04763, Republic of Korea

**Keywords:** curcumin, oxidative stress, NLRP3 inflammasome

## Abstract

**Background/Objectives**: Oxidative stress is the major cause of retinal pigment epithelial cell death. We used oxidative stress-injured retinal pigment epithelial cells to investigate the protective effects of curcumin, a strong antioxidant, on the Nod-like receptor protein 3 (NLRP3) inflammasome pathway. **Methods**: To evaluate the effect of curcumin, cell viability was measured with cell counting kit-8 and lactate dehydrogenase assays. Hydrogen peroxide (H_2_O_2_)-injured ARPE-19 cells were treated with different curcumin concentrations. We performed a wound healing assay and dichlorodihydrofluorescein diacetate staining. Western blotting and immunofluorescence staining were performed to evaluate the changes in inflammasome levels in the ARPE-19 cells. **Result:** H_2_O_2_ (300 μM) reduced the viability of ARPE-19 cells. However, treatment with 7.5 μM curcumin enhanced ARPE-19 cell viability and reduced cell toxicity. Curcumin also reduced reactive oxygen species (ROS) levels in the H_2_O_2_-induced damaged ARPE-19 cells and attenuated the H_2_O_2_-dependent levels of the NLRP3 inflammasome and its related signaling proteins. **Conclusions:** Curcumin demonstrated protective effects against oxidative stress in retinal pigment epithelial cells by attenuating the activation of the NLRP3 inflammasome pathway. These findings suggest the therapeutic potential of curcumin as an anti-inflammatory and antioxidant agent for macular degeneration.

## 1. Introduction

Age-related macular degeneration (AMD) is one of the leading causes of irreversible vision loss among the elderly population worldwide, significantly impacting the quality of life and independence of millions of individuals [1]. The disease is broadly categorized into dry (atrophic) and wet (neovascular) forms, with dry AMD comprising 85–90% of cases [2]. Dry AMD is characterized by the accumulation of drusen and progressive retinal pigment epithelium (RPE) atrophy, whereas wet AMD involves abnormal choroidal neovascularization that can rapidly impair central vision. The introduction of anti-vascular endothelial growth factor (anti-VEGF) therapy has significantly improved outcomes in wet AMD [3,4]; however, no effective treatment is available for dry AMD. Current management remains limited to behavioral modifications and monitoring of disease progression. Thus, there is an urgent need to develop novel therapeutic approaches for dry AMD.

Pathophysiological studies have demonstrated that dry AMD arises from complex interactions of genetic predisposition, aging, and environmental factors, which converge on common mechanisms such as oxidative stress, chronic inflammation, and mitochondrial dysfunction [5,6]. Among these, oxidative stress in RPE cells plays a central role. The RPE is especially vulnerable due to its high metabolic demands, ongoing phagocytosis of photoreceptor outer segments, and constant exposure to light and oxygen [7]. As a monolayer of highly metabolically active cells crucial for sustaining photoreceptor health, the RPE is particularly prone to oxidative stress. This susceptibility is due to its high mitochondrial load, phagocytosis of photoreceptor outer segments, and chronic exposure to light and oxygen [8,9]. Over time, the accumulation of oxidative damage leads to impaired RPE function, promoting drusen formation, inflammation, and eventual cell death. Persistent oxidative stress impairs mitochondrial function and cellular homeostasis, ultimately leading to inflammation, drusen formation, and RPE degeneration.

At the molecular level, oxidative stress activates the Nod-like receptor protein 3 (NLRP3) inflammasome, a cytosolic multiprotein complex that facilitates the maturation of caspase-1 and the subsequent release of interleukin (IL)-1β and IL-18 [10]. Increasing evidence indicates that NLRP3 inflammasome activation in RPE cells contributes to AMD progression via inflammation-induced cellular injury [11]. Moreover, drusen components can trigger inflammasome activation [12], further underscoring their pathogenic relevance. These findings position the NLRP3 inflammasome as an attractive therapeutic target for AMD and related neuroinflammatory disorders. Given the central role of inflammation and oxidative stress in dry AMD, targeting the NLRP3 inflammasome pathway represents a promising therapeutic strategy. In particular, naturally derived compounds with anti-inflammatory and antioxidant properties have gained interest due to their potential for long-term use and relatively low toxicity profiles. One such compound is curcumin, a polyphenolic compound derived from the rhizome of Curcuma longa, commonly used as a spice in turmeric and curry powders.

Naturally derived compounds with antioxidant and anti-inflammatory activities have been widely investigated as candidates for long-term use in treating chronic retinal diseases. Among them, curcumin has shown pleiotropic effects by modulating oxidative stress, inflammatory signaling, and apoptotic pathways [13]. Curcumin has been reported to downregulate nuclear factor κB (NF-κB), attenuate reactive oxygen species (ROS) generation, and protect mitochondrial function [14]. In diabetic retinopathy models, it prevented microvascular damage and neuroinflammation [15]. However, the role of curcumin in regulating the NLRP3 inflammasome in the retina remains insufficiently characterized.

AMD pathogenesis involves the convergence of oxidative and inflammatory processes with NLRP3 playing a critical role. Curcumin is a promising yet underexplored modulator of these pathways. Therefore, this study aimed to investigate the effects of curcumin on the NLRP3 inflammasome in oxidatively stressed RPE cells, providing novel insights into its therapeutic potential for dry AMD.

## 2. Materials and Methods

### 2.1. ARPE-19 Cell Culture

ARPE-19 cells were obtained from the American Type Culture Collection (Manassas, VA, USA). The cells were cultured in Dulbecco’s modified eagle medium/F-12 (DMEM/F12, Gibco, Grand Island, NY, USA) supplemented with 10% fetal bovine serum (FBS, Gibco, Grand Island, NY, USA) and 1% penicillin/streptomycin (Gibco, Grand Island, NY, USA). The cultures were maintained until about 24–30 passages at 37 °C in a humidified 5% CO_2_ incubator. Cells at 70–80% confluence were subcultured. Before treatment, cells were seeded overnight in complete medium to allow for proper attachment and recovery.

### 2.2. Cell Counting Kit (CCK)-8 Assay

To assess the protective effects of curcumin against oxidative stress-induced cytotoxicity, a Cell Counting Kit-8 (CCK-8, Dojindo, Kumamoto, Japan) assay was performed. ARPE-19 cells were seeded in 96-well plates (8 × 10^3^/well) and cultured in DMEM with 10% FBS for 24 h. Curcumin (Sigma, St. Louis, MO, USA) was prepared by dissolving in DMSO. The cells were treated with curcumin, at concentrations of 1–30 μM for 24 h and then additionally treated with 300 μM hydrogen peroxide (H_2_O_2_) for 16 h. After washing, 100 μL of serum-free media and 10 μL of the CCK-8 reagent (Dojindo, Kumamoto, Japan) were added to each well. After 2 h of incubation, cell viability was assessed using an enzyme-linked immunoassay (ELISA) plate reader (Thermo Fisher, Cleveland, OH, USA) at 450 and 650 nm.

### 2.3. 3-(4,5-Dimethylthiazol-2-yl)-2,5-diphenyl-2H-tetrazolium Bromide (MTT) Assay

A total of 8 × 10^3^ cells were seeded in a 96-well plate and treated with curcumin at concentrations of 1–30 μM for 24 h and 300 μM H_2_O_2_ for 16 h. The cells were washed twice and then suspended in a medium with 100 μL of 1 mg/mL thiazolyl blue tetrazolium bromide (Sigma, St. Louis, MO, USA). After 2 h of incubation, the cells were removed from the solution and placed in 200 μL dimethyl sulfoxide (Panreac, Barcelona, Spain). The plates were resuspended on a microplate mixer for 8 min without light and analyzed using an ELISA reader at 540 nm.

### 2.4. Lactate Dehydrogenase (LDH) Assay

After treatment with curcumin (1–30 μM) for 24 h, 8 × 10^3^ cells were exposed to 300 μM H_2_O_2_ for 16 h in a 96-well plate. The treated ARPE-19 cells were centrifuged at 1000 rpm for 10 min at 27 °C. Subsequently, the supernatant was moved to a new 96-well plate, colorimetric solutions (Roche, Indianapolis, IN, USA) were added, and the mixture was incubated for 30 min without light according to the manufacturer’s instructions. Cytotoxicity was assessed using an ELISA reader at 492 and 690 nm.

### 2.5. Dichlorodihydrofluorescein Diacetate (DCF-DA) Staining

To measure ROS production, the ARPE-19 cells (4 × 10^5^/well) treated for 24 h with 5 and 7.5 μM of curcumin were exposed to 300 μM H_2_O_2_ for 2 h in a six-well plate. After incubation with 10 μM 2′,7′-dichlorodihydrofluorescein diacetate (Molecular Probes, Eugene, OR, USA) at 37 °C for 15 min, the cells were washed thrice with phosphate-buffered saline (PBS). Accumulation of dichlorodihydrofluorescein in cells was assessed using a fluorescence microscope at the appropriate excitation and emission wavelengths. All results relative to ROS generation were normalized to the number in phase contrast.

### 2.6. Wound Healing Assay

In total, 1 × 10^7^ cells were added to a six-well plate. When the ARPE-19 cells had grown to 95%, they were wounded by scratching each wall with a sterile tip. They were treated with a medium containing curcumin (5 and 7.5 μM) for 24 h, and then treated with 300 μM H_2_O_2_ for 24 h. Photographs were taken of the selected regions using a phase-contrast microscope. Pictures were captured again in the same regions after another 24 h had elapsed. Wound closure was quantified using ImageJ software (1.53k ver.) by measuring the area of cell-free zones.

### 2.7. Immunofluorescence

To assess the effects of 300 μM H_2_O_2_ and curcumin on the NLRP3 inflammasome pathway of the ARPE-19 cells, the cells were seeded at a concentration of 1 × 10^5^ cells on a four-well slide glass plate, treated with curcumin (5 and 7.5 μM), and exposed to H_2_O_2_ for 2 h. They were then fixed with 2% paraformaldehyde in Dulbecco’s phosphate-buffered saline (DPBS, Gibco, Grand Island, NY, USA) for 15 min, permeabilized with 0.5% Triton X-100 in DPBS for 5 min, and washed several times with DPBS for 5 min. Next, the cells were incubated with DPBS supplemented with 5% bovine serum albumin (BSA, Millipore, Billerica, MA, USA) for 60 min.

Then, primary antibodies were added for overnight incubation at 4 °C. All the primary antibodies were diluted as follows: rabbit anit-NLRP3 (1:100, #NBP2-12446, NOVUS Bio. Littleton, CO, USA) and rabbit anti-IL-1β (1:100, #ab9722, Abcam, Cambridge, MA, USA). Cell nuclei were stained with 4′,6-diamidino-2-phenylindole (Vector Laboratories, Burlingame, CA, USA). Secondary antibodies, including goat anti-rabbit 488 and goat anti-mouse Alexa Fluor 633 (Invitrogen, Carlsbad, CA, USA), were diluted in 5% BSA. Images were acquired using an Olympus microscope (Olympus, Tokyo, Japan).

### 2.8. Western Blotting

The ARPE-19 cells (2 × 10^5^/well) treated with 5 and 7.5 μM of curcumin were exposed to 300 μM H_2_O_2_ for 2 h in a six-well plate. The cells were washed twice in cold PBS and incubated for 30 min on ice in a RIPA lysis buffer (Sigma) and protease inhibitor (Thermo Fisher, Cleveland, OH, USA). Harvested the cell suspension was sonicated five to ten times using a Sonoplus apparatus on ice. The samples were centrifuged at 13,000 rpm for 15 min at 4 °C.

Western blotting analysis was performed as previously described [16]. The primary antibodies used in this study were as follows: rabbit anti-NLRP3 (1:500, #NBP2-12446, NOVUS Bio. Littleton, CO, USA), rabbit anti-IL-1β (1:500, #ab9722, Abcam, Cambridge, MA, USA), rabbit anti-caspase-1 (1:500, #NBP1-45433, NOVUS Bio. Littleton, CO, USA), β-actin (1:2000, #4967S, Cell Signaling Technology, Beverly, MA, USA). After washing, the membranes were incubated with horseradish peroxidase-conjugated anti-rabbit antibody (#711-035-152, Jackson ImmunoResearch Laboratories Inc., West Grove, PA, USA) and visualized using enhanced chemiluminescence (Thermo Fisher, Cleveland, OH, USA) using appropriate detection equipment.

### 2.9. Statistical Analyses

All experimental data were obtained from at least two technical replicates, and each experiment was performed independently 3–5 times. Data are presented as mean ± standard deviation (SD) from independent biological replicates (n = 3–5). Statistical analyses were performed using GraphPad Prism 8 (GraphPad Software, San Diego, CA, USA). Normality of the data was assessed using the Shapiro–Wilk test, and one-way analysis of variance (ANOVA) followed by Tukey’s post hoc test was employed to determine statistical differences between groups. A *p*-value less than 0.05 was considered statistically significant. The significance levels were marked as follows: * *p* < 0.05, ** *p* < 0.01, and *** *p* < 0.001.

## 3. Results

### 3.1. Effect of Oxidative Stress and Curcumin on ARPE-19 Cell Viability

**Figure 1:**
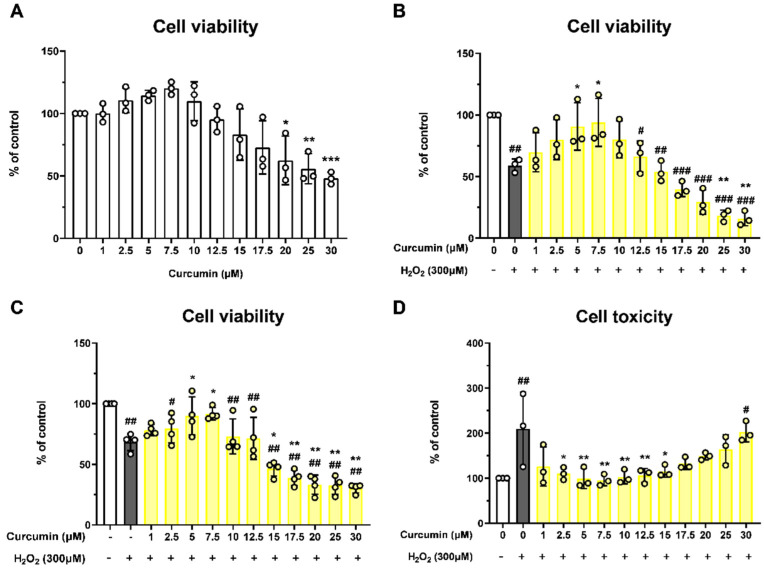
The effect of curcumin (0–30 μM; dimethyl sulfoxide, 0.5%) on the cell viability of ARPE-19 cells. Treatment of >20 μM curcumin significantly reduced ARPE-19 cell viability (**A**). Several concentrations of curcumin and 300 μM H_2_O_2_ were applied, and the cell viability was measured using a cell counting kit-8 (**B**), and an MTT assay (**C**). 5 and 7.5 μM curcumin showed protective effects in both experiments. Cell cytotoxicity was measured using a lactate dehydrogenase assay (**D**). Cell toxicity was reduced by curcumin treatment (2.5–15 μM). Data are presented as the mean ± standard deviation (SD) from 3 to 4 independent experiments, each performed in duplicate. Statistical analysis was performed using one-way analysis of variance. * *p* < 0.05, ** *p* < 0.01, *** *p* < 0.001, (H_2_O_2_ + 0 μM curcumin group) vs. # *p* < 0.05, ## *p* < 0.01, ### *p* < 0.001. (Normal group).

The effects of oxidative stress and curcumin on ARPE-19 cell viability were evaluated using CCK, MTT, and LDH assays. Cells were treated with various concentrations of curcumin to assess cytotoxicity and viability (Figure 1A). High concentrations of curcumin (20–30 μM) significantly reduced cell viability, indicating cytotoxic effects. In contrast, treatment with 5 and 7.5 μM curcumin significantly improved cell viability in cells exposed to 300 μM H_2_O_2_, compared to H_2_O_2_-treated controls (Figure 1B,C). Moreover, curcumin at concentrations ranging from 2.5 to 15 μM effectively reduced cytotoxicity, as shown by LDH assay results (Figure 1D). Based on these findings, 5 and 7.5 μM curcumin were selected for subsequent experiments.

### 3.2. Effect of Curcumin on ROS Production

**Figure 2:**
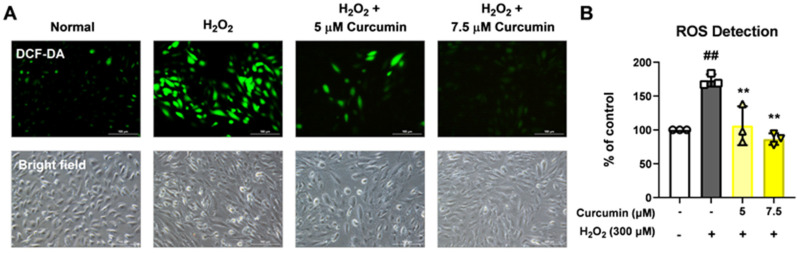
The effect of curcumin (5 and 7.5 μM) on reactive oxygen species (ROS) measurement of ARPE-19 cells. The cells were treated with 5 and 7.5 μM of curcumin and 300μM H_2_O_2_. ROS measurement was demonstrated by dichlorodihydrofluorescein diacetate (DCF-DA) staining (**A**). Treatment with 5 and 7.5 μM curcumin significantly downregulated ROS fluorescence signaling (**B**). Scale bar: 100 μm. Quantification was performed from 3 independent experiments (biological replicates). For each experiment, at least three fields of view were imaged and averaged (technical replicates). Data are presented as the mean ± SD. Statistical analysis was performed using one-way analysis of variance. ** *p* < 0.01, (H_2_O_2_ + 0 μM curcumin group) vs. ## *p* < 0.01, (normal group).

To investigate the antioxidant potential of curcumin, we assessed intracellular ROS levels in ARPE-19 cells with oxidative stress. Treatment with 300 µM H_2_O_2_ led to increase in ROS production, indicating elevated oxidative stress within the cells (Figure 2A). However, co-treatment with curcumin at concentrations of 5 µM and 7.5 µM significantly suppressed ROS generation. Notably, 7.5 µM curcumin reduced ROS levels, suggesting a strong ROS scavenging effect (Figure 2A,B). These results demonstrate that curcumin effectively attenuates H_2_O_2_-induced oxidative stress in RPE cells, likely through its potent antioxidant properties.

### 3.3. Effect of Curcumin on Cell Migration

**Figure 3:**
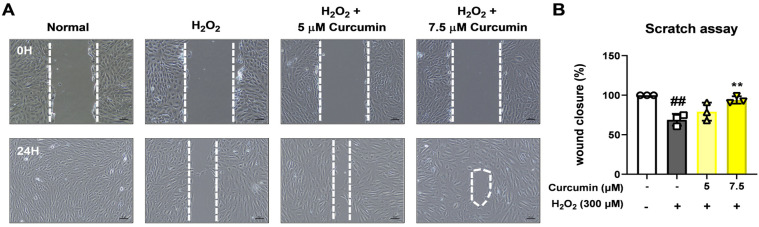
Cell migration was determined using a wound healing assay (**A**) Quantitative level of migration of ARPE-19 cells was increased in ARPE-19 cells that were treated with 7.5 μM curcumin compared with the levels that were treated with 300 μM H_2_O_2_ (**B**). Scale bar: 50 μm. Data are presented as the mean ± standard deviation (SD) from 3 independent experiments, each performed in duplicate. Statistical analysis was performed using one-way analysis of variance. ** *p* < 0.01, (H_2_O_2_ + 0 μM curcumin group) vs. ## *p* < 0.01, (Normal group).

We examined whether curcumin could support functional recovery in RPE cells under oxidative stress by evaluating cell migration. A wound healing assay was performed to assess the migratory capacity of ARPE-19 cells after H_2_O_2_-induced damage (Figure 3A). Exposure to 300 µM H_2_O_2_ significantly impaired cell migration, indicating reduced regenerative ability under oxidative stress. However, co-treatment with 5 µM and 7.5 µM curcumin substantially improved the wound closure rate, with 7.5 µM curcumin showing a more pronounced recovery in cell migration compared to the H_2_O_2_-only group (Figure 3B).

### 3.4. Effect of Oxidative Stress and Curcumin on Intracellular NLRP3 Inflammasome Pathway Protein Levels

**Figure 4:**
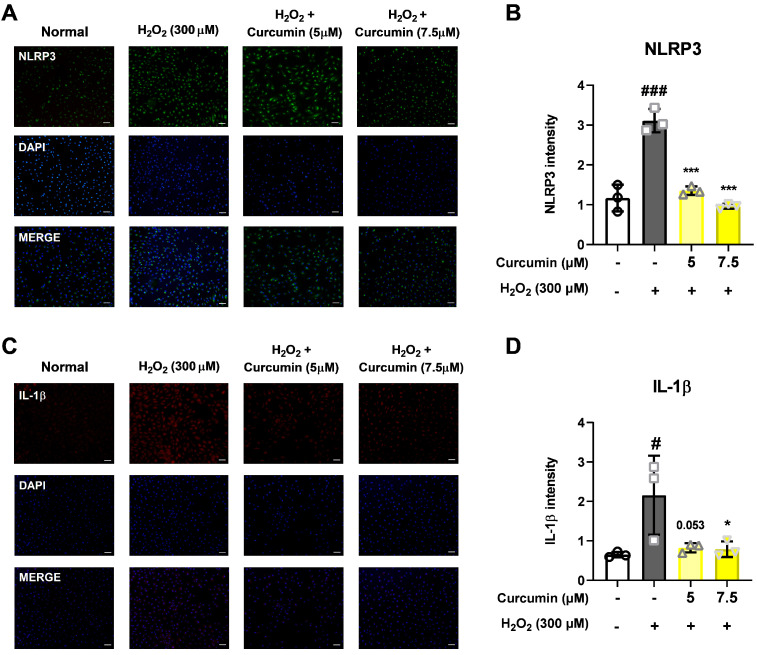
Assessment of NLRP3 inflammasome protein level and localization in ARPE-19 cells with curcumin. The cells were treated with 5 and 7.5 μM of curcumin and 300 μM H_2_O_2_, and the levels of the NLRP3 inflammasome pathway proteins were assessed by immunocytochemistry (**A**,**C**). The immunoreactivity levels of NLRP3 and IL-1β were decreased in cells that were treated with 5 and 7.5 μM curcumin compared with the levels in cells that were treated with 300 μM H_2_O_2_ (**B**,**D**). Scale bar: 100 μm. Data were obtained from 3 independent experiments (biological replicates). For each experiment, at least three fields of view were captured and averaged (technical replicates). Values are expressed as mean ± SD. Statistical analysis was performed using one-way analysis of variance. * *p* < 0.05, *** *p* < 0.001, (H_2_O_2_ + 0 μM curcumin group) vs. # *p* < 0.05, ### *p* < 0.001. (Normal group).

**Figure 5:**
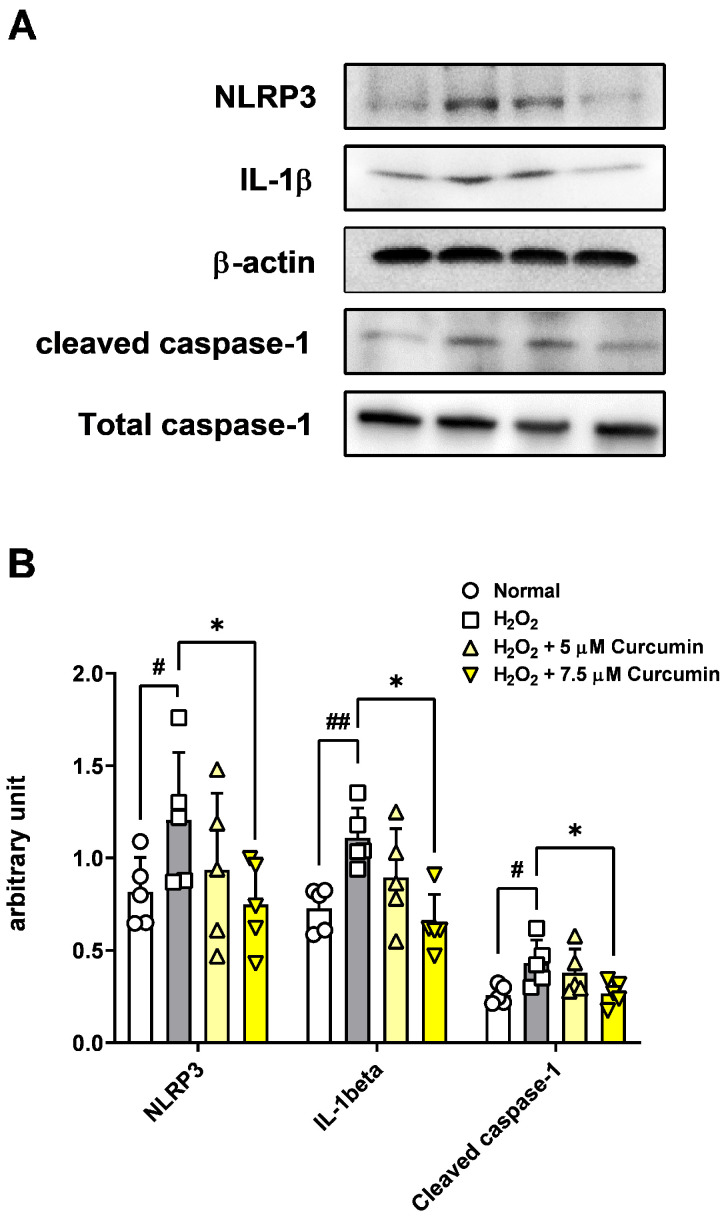
The effect of curcumin (5 and 7.5 μM) on comparing the NLRP3 inflammasome pathway levels in ARPE-19 cells. The cells were treated with 5 and 7.5 μM of curcumin and 300 μM H_2_O_2_, and the levels of the NLRP3 inflammasome pathway proteins were assessed by Western blotting (**A**). Protein level of NLRP3 and IL-1β were decreased in cells that were treated with 5 and 7.5 μM curcumin compared with the levels in cells that were treated with 300 μM H_2_O_2_ (**B**). Scale bar: 100 μm. Data are presented as the mean ± standard deviation (SD) from 5 independent experiments, each performed in duplicate. Statistical analysis was performed using one-way analysis of variance. * *p* < 0.05, (H_2_O_2_ + 0 μM curcumin group) vs. # *p* < 0.05, ## *p* < 0.01 (normal group).

The levels of inflammasome pathway-related proteins (NLRP3, IL-1β, and caspase-1) were increased in ARPE-19 cells treated with H_2_O_2_. Conversely, curcumin significantly decreased the levels of NLRP3 inflammasome pathway-related proteins. This inhibitory effect of curcumin was confirmed by immunofluorescence staining, which showed decreased protein localization (Figure 4), and was further validated by Western blot analysis demonstrating reduced protein levels (Figure 5A,B. These findings indicate that curcumin effectively suppresses oxidative stress-induced activation of the NLRP3 inflammasome pathway in RPE cells.

## 4. Discussion

This study investigated the protective effects of curcumin in retinal pigment epithelial cells exposed to oxidative stress, with a particular focus on its modulation of the NLRP3 inflammasome signaling pathway. Oxidative stress is a key pathogenic factor in many retinal degenerative diseases, including AMD, and plays a significant role in disrupting the function of RPE cells. In our model using ARPE-19 cells, exposure to 300 μM H_2_O_2_ significantly reduced cell viability and impaired cell migration while simultaneously increasing cytotoxicity, intracellular ROS levels, and the levels of key inflammasome pathway proteins, including NLRP3, caspase-1, and IL-1β. However, curcumin treatment produced notable protective effects. At concentrations of 5 and 7.5 μM, curcumin significantly restored cell viability, and in the broader range of 2.5–15 μM, it reduced cytotoxicity in a dose-dependent manner. Moreover, curcumin at 5 and 7.5 μM markedly decreased ROS production, suggesting potent antioxidant capacity. Notably, curcumin also improved the migratory ability of oxidatively stressed ARPE-19 cells, which is essential for retinal repair mechanisms. Additionally, treatment with 7.5 μM curcumin effectively downregulated the levels of NLRP3 inflammasome pathway-related proteins, indicating that its cytoprotective effects are at least partially mediated through the inhibition of inflammasome activation. Curcumin not only restored cell viability and migration capacity but also reduced ROS levels and suppressed activation of the NLRP3 inflammasome pathway.

The RPE is vulnerable to oxidative stress due to its high metabolic demand and continuous exposure to oxygen and light [17]. Functional impairment of the RPE disrupts the integrity of the outer blood–retinal barrier and initiates a cascade of neuroinflammatory responses, ultimately resulting in photoreceptor degeneration and vision loss. As oxidative damage is a central hallmark of AMD pathology, therapeutic approaches aimed at preserving RPE function under oxidative stress are of critical importance.

Curcumin is well recognized for its antioxidant, anti-inflammatory, and anti-apoptotic properties. Numerous studies have reported that curcumin can suppress NF-κB signaling, a transcription factor complex that serves as the central upstream regulator of the NLRP3 inflammasome. Based on previous studies [18], the suppression of NF-κB by curcumin reduces inflammatory gene expression and may indirectly modulate inflammasome activation. The role of curcumin in retinal diseases has been previously investigated. Studies have demonstrated that oral administration of curcumin to streptozotocin-induced diabetic rats prevents damage to retinal blood vessels and downregulates various inflammatory factors in the retina [19,20]. In addition, studies have reported that curcumin prevents apoptosis and inhibits apoptosis-related factors in retinal endothelial and RPE cells exposed to high glucose [21,22]. Furthermore, in rat models of light-induced retinal degeneration, curcumin mixed into the diet downregulated pro-inflammatory, pro-apoptotic, and oxidative stress-related genes and suppressed NF-κB to inhibit cellular inflammatory genes [23].

In the present study, treatment with curcumin, particularly at a concentration of 7.5 µM, significantly mitigated H_2_O_2_-induced oxidative stress and decreased the protein levels of NLRP3, IL-1β, and caspase-1, which are key components of the inflammasome signaling cascade in ARPE-19 cells. These findings indicate that curcumin confers cytoprotective effects, at least in part, by suppressing NLRP3 inflammasome activation under oxidative stress. This observation is consistent with prior reports in other pathological contexts, including diabetic nephropathy [24] and epilepsy [25], where curcumin has been shown to effectively inhibit NLRP3 activity, thereby strengthening its potential as a widely applicable anti-inflammatory and antioxidant agent.

Curcumin not only attenuated oxidative stress but also facilitated tissue repair in ARPE-19 cells, as evidenced by enhanced cell migration in the wound healing assay. Given that proper migration is essential for RPE cells to maintain retinal homeostasis and respond to injury, curcumin’s ability to promote this process highlights its therapeutic potential. This effect may be particularly relevant in retinal degenerative diseases, where delayed or insufficient cellular repair contributes to disease progression.

This is the first investigation to comprehensively examine the effects of curcumin on the NLRP3 inflammasome pathway in RPE cells under conditions of oxidative stress. While prior research has suggested curcumin’s potential to modulate diverse inflammatory responses in various experimental retinal disease models, none have directly and systematically investigated its specific role in suppressing NLRP3 activation within the pathological context of RPE oxidative damage. This finding is particularly significant as it provides important and novel mechanistic insights into how curcumin may confer sustained and multifaceted protective effects against progressive retinal degenerative diseases, including but not limited to AMD.

Additionally, our results suggest that the suppression of the inflammasome may be a downstream consequence of curcumin’s ability to reduce ROS accumulation in the cells. As oxidative stress is a known activator of the NLRP3 inflammasome, the dual action of curcumin—both as a ROS scavenger and an inflammasome inhibitor—may confer a synergistic benefit. In addition, curcumin’s effects on NF-κB and IκBα levels may further link oxidative stress and inflammation regulation, providing a unifying mechanism of action [26]. Several studies have confirmed that curcumin inhibits NF-κB translocation to the nucleus, thereby reducing the transcription of inflammatory genes [27,28,29]. Since NF-κB serves as a bridge between oxidative stress and inflammation, curcumin’s inhibition of this pathway may simultaneously reduce oxidative damage and prevent chronic inflammation in the retina. Since NF-κB is a key upstream regulator that promotes the transcription of NLRP3 and other pro-inflammatory cytokines, curcumin’s inhibition of NF-κB signaling may underlie its suppressive effects on NLRP3 activation. This suggests a potential mechanistic link through which curcumin mitigates oxidative stress-induced inflammation in RPE cells.

The therapeutic implications of the present findings are not confined solely to AMD. Persistent oxidative stress and aberrant inflammasome activation have been implicated in a wide spectrum of retinal and neurodegenerative disorders, including diabetic retinopathy, glaucoma, and Alzheimer’s disease, all of which exhibit convergent pathological hallmarks, such as mitochondrial dysfunction, excessive accumulation of ROS, and sustained activation of innate immune pathways. The ability of curcumin to concurrently modulate oxidative stress and inflammatory signaling underscores its potential as a therapeutic candidate for multifactorial degenerative conditions. Curcumin primarily reduces the upstream triggers of oxidative stress, such as ROS, which in turn indirectly modulate downstream effectors, including the NLRP3 inflammasome, IL-1β, and caspase-1. Accordingly, our findings suggest that curcumin exerts protective effects against oxidative stress in RPE cells by attenuating NLRP3 inflammasome activation through the reduction in ROS levels, highlighting its potential translational relevance. Furthermore, considering the pleiotropic nature of curcumin’s actions, future investigations should delineate its synergistic interactions with other antioxidants or inflammasome inhibitors, thereby providing a more comprehensive framework for the development of novel therapeutic interventions in retinal and neurodegenerative diseases.

This study has several limitations. First, the experiments were conducted in vitro using ARPE-19 cells, which, although widely used, may not fully recapitulate the behavior of primary RPE cells or RPE cells in vivo. Further in vivo studies using animal models of AMD are essential to validate the protective role of curcumin and its modulation of the NLRP3 inflammasome pathway in a physiologically relevant environment. Additionally, we demonstrated in cells that the NLRP3 inflammasome pathway is regulated by curcumin; however, the mechanisms regulating this pathway have not been investigated. Another major challenge in translating curcumin for clinical use is its poor bioavailability. Curcumin is characterized by low water solubility, rapid metabolism, and poor systemic absorption upon oral administration. Curcumin’s clinical translation has been limited by poor systemic bioavailability, prompting the development of nanoparticles, liposomes, and phospholipid formulations to enhance tissue delivery [30]. Future research should investigate whether advanced formulations can achieve effective concentrations in the retina and exert protective effects similar to those observed in our in vitro model. Furthermore, oxidative stress activates not only the NLRP3 inflammasome pathway but also networks of interconnected signaling cascades. These include the JNK, p38 MAPK, and PI3K/AKT pathways, which contribute to inflammation, apoptosis, and cellular senescence in RPE cells. Other well-established markers, including deoxyribonucleic acid (DNA) fragmentation, protein dysfunction, malondialdehyde (MDA), glutathione/glutathione disulfide (GSH/GSSG), superoxide dismutase (SOD), and catalase (CAT) were not examined. The evaluation of these parameters would provide a more comprehensive understanding of the antioxidant defense system. Future investigations are warranted to incorporate these additional assays to validate and extend the present findings. Elucidating whether curcumin modulates these parallel pathways will be critical for a comprehensive understanding of its protective functions and therapeutic potential in AMD.

Therapeutic strategies that concurrently address multiple pathological mechanisms are of considerable significance. Curcumin has been shown to attenuate oxidative stress, inhibit inflammasome activation, and suppress NF-κB signaling, thereby supporting its potential as a multi-target therapeutic candidate. In addition, its favorable safety profile, documented through extensive use in traditional medicine and supported by numerous preclinical investigations, further substantiates its suitability for clinical use. To fully realize its therapeutic potential, future research should investigate combination approaches in which curcumin is administered in conjunction with other antioxidants or anti-inflammatory agents, or incorporated into advanced nanocarrier systems engineered to enhance its bioavailability and retinal delivery. Such strategies may augment therapeutic efficacy while mitigating systemic adverse effects, thereby providing a more comprehensive framework for the management of retinal degenerative disorders, such as AMD.

## 5. Conclusions

Our findings demonstrate that curcumin (7.5 μM) reduces ROS level, which subsequently attenuates NLRP3 inflammasome activation, thereby exerting a protective effect against oxidative stress in RPE cells. These results suggest that curcumin has therapeutic potential as an anti-inflammatory and antioxidant agent in AMD. Further preclinical and clinical investigations are warranted to confirm its efficacy and optimize its delivery to retinal tissues.

## Data Availability

The original contributions presented in this study are included in the article. Further inquiries can be directed to the corresponding author(s).
